# Phasor analysis of NADH FLIM identifies pharmacological disruptions to mitochondrial metabolic processes in the rodent cerebral cortex

**DOI:** 10.1371/journal.pone.0194578

**Published:** 2018-03-21

**Authors:** Carlos A. Gómez, Jason Sutin, Weicheng Wu, Buyin Fu, Hana Uhlirova, Anna Devor, David A. Boas, Sava Sakadžić, Mohammad A. Yaseen

**Affiliations:** 1 Athinoula A. Martinos Center for Biomedical Imaging, Department of Radiology, Massachusetts General Hospital, Harvard Medical School, Charlestown, MA, United States of America; 2 Department of Neurosciences and Radiology, UC San Diego, La Jolla, CA, United States of America; 3 Central European Institute of Technology, Brno University of Technology, Brno, Czech Republic; University of California Berkeley, UNITED STATES

## Abstract

Investigating cerebral metabolism *in vivo* at a microscopic level is essential for understanding brain function and its pathological alterations. The intricate signaling and metabolic dynamics between neurons, glia, and microvasculature requires much more detailed understanding to better comprehend the mechanisms governing brain function and its disease-related changes. We recently demonstrated that pharmacologically-induced alterations to different steps of cerebral metabolism can be distinguished utilizing 2-photon fluorescence lifetime imaging of endogenous reduced nicotinamide adenine dinucleotide (NADH) fluorescence *in vivo*. Here, we evaluate the ability of the phasor analysis method to identify these pharmacological metabolic alterations and compare the method’s performance with more conventional nonlinear curve-fitting analysis. Visualization of phasor data, both at the fundamental laser repetition frequency and its second harmonic, enables resolution of pharmacologically-induced alterations to mitochondrial metabolic processes from baseline cerebral metabolism. Compared to our previous classification models based on nonlinear curve-fitting, phasor–based models required fewer parameters and yielded comparable or improved classification accuracy. Fluorescence lifetime imaging of NADH and phasor analysis shows utility for detecting metabolic alterations and will lead to a deeper understanding of cerebral energetics and its pathological changes.

## Introduction

The electrochemical signaling that takes place between neurons, glia, and cerebral microvasculature during healthy brain function is extraordinarily complex and energetically demanding. To maintain this intricate, relentless activity, the brain is vitally dependent on a well-regulated, uninterrupted supply of metabolites from the bloodstream and continuous oxidative metabolism within neurons and glia [1]. For many pathological conditions, including neurodegenerative diseases, cancer, and stroke, alterations in cerebral energy metabolism constitute a hallmark of disease onset or advancement [2,3]. Such alterations, including relative shifts from oxidative to glycolytic metabolism, reduced mitochondrial membrane potential, and oxidative stress, manifest with the onset and progression of these disorders and are often associated with impairments of blood flow and metabolite supply [4–7]. Understanding these disease-related variations is essential for development of novel biomarkers and therapeutic targets.

Among existing technologies for investigating cerebral metabolism, 2-photon microscopy (2PM) uniquely enables minimally invasive observation of multiple facets of blood flow and energy metabolism at cellular and subcellular resolutions *in vivo*, where the structural and functional connections remain undisturbed. For *in vivo* imaging, 2PM offers distinct advantages over other methods such as ultrasound, X-ray computed tomography, magnetic resonance imaging, and positron emission tomography. Benefits include high axial and lateral resolutions, deeper optical penetration relative to single-photon based confocal microscopy, and broader 2-photon absorption spectra of many common fluorophores, which enables simultaneous excitation of multiple fluorophores with distinct emission spectra using a single excitation wavelength. [8–10]. Applying 2PM for measuring endogenous fluorescence of reduced nicotinamide adenine dinucleotide (NADH) has demonstrated utility as a minimally invasive technique for evaluating metabolic activity [11,12]. NADH is ubiquitous within tissues and functions as the principal electron carrier during breakdown of glucose and other metabolites in both anaerobic glycolysis and aerobic oxidative metabolism [12]. Both oxidized NAD^+^ and reduced NADH show strong absorption in the UV region around 260 nm; however, only NADH absorbs appreciably at 350–365 nm and emits fluorescence with a peak at ~460 nm. The emitted fluorescence of NADH serves as a useful endogenous, nondestructive marker for variations in metabolic activity [4,13]. Using fluorescence lifetime imaging (FLIM), multiple enzyme-bound formulations of NADH can reportedly be resolved through multi-exponential fitting of time-resolved fluorescence profiles [14,15]. Reports vary regarding the total number of enzyme-bound formulations, or species, of endogenous NADH that can be observed with FLIM and their respective roles in different processes of metabolism. The shortest-lifetime species is widely agreed to correspond to ‘free’ NADH, while longer-lifetime species ostensibly represent fluorescence contributions from multiple enzyme- and protein- bound formulations of NADH and the spectrally- identical biosynthesis co-factor NADPH [16,17].

We previously coupled 2PM and FLIM to explore the metabolic significance of multiple NADH species for evaluating metabolic activity *in vivo* in the rat cortex [18,19]. After developing a gravity-feed perfusion system and integrating it into our sealed-cranial window preparation, we delivered well-characterized pharmacological modulators of anaerobic glycolysis and aerobic oxidative metabolism to the exposed cortical surface. We observed that, although the NADH species are not directly relatable to specific enzymes or proteins, FLIM observations of cerebral NADH could resolve variations from baseline activity through alterations in glycolysis, the Krebs’ cycle, oxidative phosphorylation, and respiration. As a representative pathological condition that disrupts cerebral metabolism, we induced focal seizure activity by administering 1 mM bicuculline-methiodide (BMI), a disinhibiting antagonist of receptors for gamma-aminobutyric acid-A [20]. We developed classification models that identified seizure-associated metabolic alterations as disruptions to the electron transport chain, likely induced by deficits in oxygen supply.

**Fig 1:**
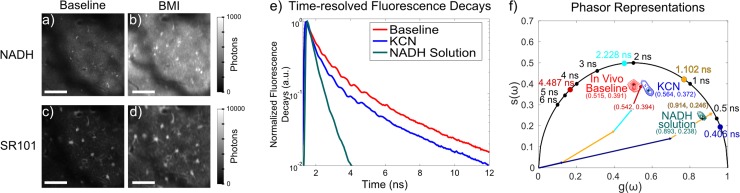
(a-d) Representative fluorescence intensity images of (a,b) endogenous NADH fluorescence and (c,d) the topically-applied astrocyte label sulforhodamine 101 in the anesthetized rat cerebral cortex. Imaging was performed (a,c) before and (b,d) after BMI-induced focal seizure activity, Scale bar: 50 μm (e) Time-resolved fluorescence decays for NADH in solution (teal) and (red) in the rat cortex before pharmacological inhibition and after KCN (blue) administration. Fluorescence decays were measured at each pixel in the images and used for phasor computations. (f) Corresponding phasor contour representations of the same data, with peak phasor coordinates listed. Colored circles indicate previously-computed lifetime values of cortical NADH. Solid arrows represent the fractionally-weighted vectorial contribution of each lifetime to the phasor measurement.

In this report, we applied *phasor*, or *polar plot*, -based computations to our previously-collected dataset of pharmacological inhibitions, to assess the phasor method’s prospective advantages over conventional nonlinear curve fitting. Nonlinear curve-fitting analysis is computationally-intensive and sensitive to potential inaccuracies due to correlations between computed terms. Conversely, phasor analysis provides a computationally-simple, robust approach to investigate variations in fluorescence lifetime measurements [21–23]. Additionally, it permits easy visualization of molecular interactions and microenvironmental changes that alter fluorescence lifetime as clusters on the 2D polar plot representation of computed phasor coordinates (Fig 1). The technique is particularly well-suited for Förster resonance energy transfer (FRET) studies involving multiple fluorophores with well-pronounced differences in donor and acceptor lifetimes. We assessed the ability of the phasor method for evaluating alterations in cerebral metabolism observed with NADH FLIM. To assess the feasibility of the phasor method and compare its performance to nonlinear modeling, we developed classification models and evaluated their ability to classify the BMI-induced seizure activity as disruptions to the electron transport chain. We compared the models’ performance to those developed with our previously-calculated nonlinear curve-fitting data. We demonstrate that pharmacological manipulations of mitochondrial metabolism induce resolvable shifts of the NADH phasor representation on the 2D polar plot. Compared to our previous nonlinear-fitting analyses, the phasor method enabled development of statistical classification models with fewer parameters and lower cross-validation error that yielded similar or better ability to predict metabolic disruptions associated with BMI-induced seizure activity.

## Materials and methods

**Fig 2:**
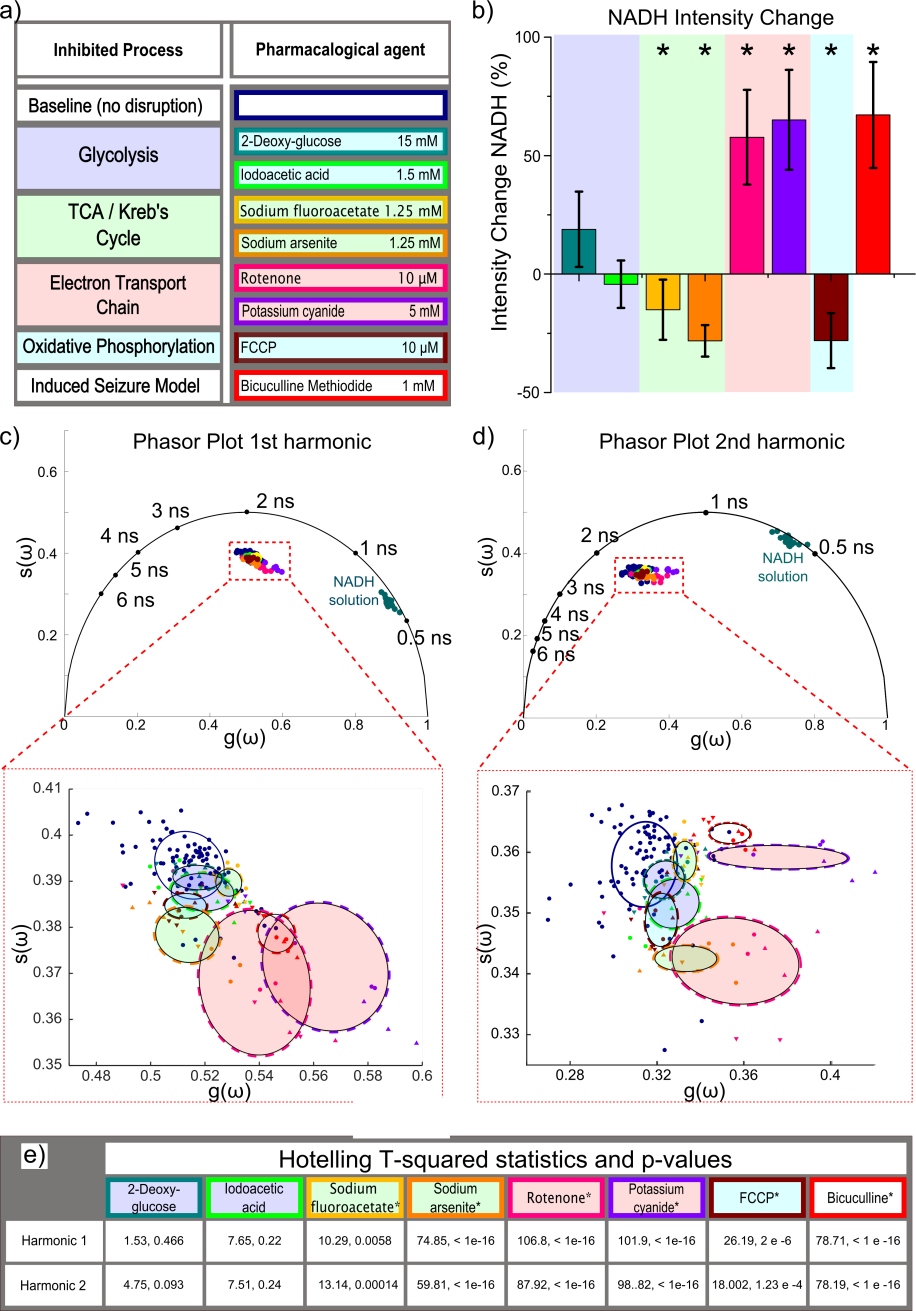
(a) Pharmacological agents categorized by affected metabolic process, color-coded for subsequent panels (b) Induced changes to NADH intensity relative to baseline levels in pixels corresponding to astrocytic cell bodies and neuropil. * indicates statistically significant variation from baseline (c)Phasor plots for cortical NADH after application of different pharmacological inhibitors at the fundamental laser repetition frequency (ω = 2π × 80 MHz) and (c) second harmonic (ω = 4π × 80 MHz). Lower insets zoomed in to show standard deviational ellipses of each pharmacological reagent, along with data points used to compute ellipses. Ellipse boundary and data points are colored to correspond with reagents in (a). Each data point shape corresponds to a different animal.

*In vivo* imaging experiments were conducted under a protocol approved by the Institutional Animal Care and Use Committee at Massachusetts General Hospital. Experimental data acquisition was described previously when these measurements were analyzed using nonlinear analysis [19]. Succinctly, using the 2PM portion of a custom-designed multimodal imaging system [24] coupled with Time-Correlated Single Photon Counting (TCSPC) acquisition hardware [14], FLIM measurements of intrinsic NADH fluorescence were collected *in vivo* from surgically-exposed cortices of Sprague Dawley rats [10] before and after locally delivering [25] well-characterized inhibitors of different processes of glycolysis and oxidative metabolism (Fig 2A). Using the perfusion system, approximately 1–2 ml of the pharmacological agents dissolved in artificial cerebrospinal fluid were delivered to the brain surface at a rate not exceeding 0.5 ml/min. Additional time was allowed for the reagents to diffuse into the cortex. The time between baseline measurements and measurements after metabolic perturbation ranged from 10–30 minutes. Incident power for excitation (from Mai Tai laser, Spectra Physics, 80MHz, ~360 fs at the back focal aperture, λ_ex_: 740 nm, λ_em_: 460 ± 30 nm) was limited well below 50 mW at the samples, yielding photon rates of ~125,000 counts/s. A water-immersion high numerical aperture objective (Olympus XLumPlan Fluor, 20X, 1.00 NA, 2 mm working distance) was used to image over ~200 μm fields of view. Acquisitions lasted 2 minutes with ~1 fps frame rate and 6.4 μs pixel dwell time. During surgical preparation, astrocyte-specific sulforhodamine 101 (SR101) [26] was topically applied, providing morphological guidance during image acquisition.

Custom software was developed in MATLAB to process our NADH FLIM data using the phasor analysis method [23,27]. A 3x3 median filter was applied to each image [27]. Pixels corresponding to blood vessels were masked out manually. For the remaining pixels representing extravascular tissue, time-resolved fluorescence decay profiles were binned with their neighboring pixels (3x3 pixel binning), yielding a minimum of 5000 photons per pixel. A fluorescence standard (1.5 mM NADH dissolved in phosphate-buffered saline) was measured before each experiment and used for our calibration procedure for phasor computation, as described in the supplementary information. For each pixel, experimental phasor coordinates, g(ω) and s(ω), were computed as the intensity-normalized real and imaginary components of the fluorescence decay’s Fourier transform at radial frequency ω = 2πf (where f is the laser repetition frequency 80 MHz) [23,28].

$$g_{i,j}(\omega )=\frac{\int_{0}^{\infty }I_{i,j}(t)\cos(\omega t)dt}{\int_{0}^{\infty }I_{i,j}(t)dt}$$

$$s_{i,j}(\omega )=\frac{\int_{0}^{\infty }I_{i,j}(t)\sin\left(\omega t\right)dt}{\int_{0}^{\infty }I_{i,j}(t)dt}$$

For our acquisitions at each pixel, TCSPC measurement of fluorescence decays consisted of 12.5 ns long decays resolved into 256 timepoints. At very early timepoints, nonlinearity in the time-to-amplitude conversion circuitry (TAC) limits accurate time-correlation measurements of emitted photons, and consequently, the TCSPC hardware truncates part of the fluorescence decay. Our phasor computations were performed as done in Chen et al [28], over the entire 256-point decay measurements, including the early non-linearity period. As described in the results, our phasor computations appear in locations consistent with published findings after performing the calibration procedure (see supplementary information).

Measurements were grouped based upon their corresponding pharmacological reagent. To determine whether other values of ω permitted better separation of phasor clusters [22,27,29], our analysis also included phasor computations at integral harmonics of the laser repetition frequency. For each applied inhibitor, Hotelling t-squared statistical analysis was used to determine whether mean values measured after inhibition differed significantly from baseline conditions [30].

As in our previous report using results of nonlinear fitting [19], statistical classification models were created to assess the phasor method’s utility for precisely characterizing disruptions to cerebral metabolism. Using routines from the Statistics Toolbox in Matlab, computed data were categorized into different classes: inhibitors of either the tricarboxylic acid (TCA) cycle, the electron transport chain (ETC), or uncoupling of oxidative phosphorylation (OXPHOS). For each measurement, pixel-averaged values for g(ω), s(ω), and changes in fluorescence intensity were used as training data for classification models based on Linear Discriminant Analysis (LDA), K-nearest neighbors (KNN), or naïve Baye's (NB) classification algorithms. The quality of the models was evaluated by repeatedly isolating a subset of the training data to serve as test data and determining the K-fold cross validation error [31]. The models were applied to predict the type of metabolic inhibition induced by focal seizure experiments. Data collected with inhibitors of glycolysis were not incorporated into the classification models due to a lack of statistically significant variation from baseline, as detailed in the results.

## Results and discussion

Fig 1A–1E illustrate the alterations of cortical NADH observable by 2PM-based FLIM. Pharmacalogically-induced manipulation provoked variations in the prevalence and enzymatic binding conditions of NADH that modulate NADH fluorescence intensity (Fig 1A–1D) and its time-resolved decay (Fig 1E). Fig 1F displays contour plots of the computed phasor coordinates of NADH in solution (free NADH) and NADH measured *in vivo*. The latter observations include measurements from cortical tissue of anesthetized rats under baseline conditions (before pharmacological inhibition) and after blocking oxidative phosphorylation by applying potassium cyanide (KCN) to inhibit complex IV (cytochrome c oxidase). KCN-induced impairment to electron transport results in accumulation of free NADH by preventing its oxidation to NAD^+^, which yields observable increases in NADH intensity and reduction in average NADH lifetime. As seen in Fig 1F, a large separation exists between *in vivo* cerebral NADH measurements and NADH solution, consistent with previous NADH phasor observations *in vitro* [27], and reflective of the enzyme-bound predominance of endogenous NADH fluorescence signal from living cells and tissue. In various reports, FLIM-based NADH observations of cell cultures and tissues manifest on different regions within the boundary of the semicircular region from 0 ≤ g(ω) ≤ 1 and 0 ≤ s(ω) ≤ 0.5, commonly known as the phasor ‘universal circle’ [23,27,32,33]. Our *in vivo* observations of cerebral NADH appear to localize in the same region as *in vitro* observations of neurons and neural progenitor stem cells by Stringari et al in [34]. The plot also illustrates that these phasor computations agree with our previous characterizations involving nonlinear curve-fitting [18,19]. As previously described [22,29], phasor coordinates for fluorophores whose decay profiles follow single-exponential kinetics lie upon the semi-circular boundary of the phasor plot universal circle. Fluorophores with more complex, multi-exponential decays yield phasors that lie within the universal circle, at locations corresponding to the fractionally-weighted vectorial sum of each exponential component. In our previous characterizations involving multi-exponential curve-fitting analysis, FLIM observations for free NADH solution were best modeled as a bi-exponential decay [35], and our *in vivo* cerebral NADH measurements were best modeled as the sum of 4 exponential terms, of which the shorter 2 lifetimes were fixed at values computed for free NADH. Colored points along the ‘universal circle’ in Fig 1F correspond to the lifetime values computed in our previous analysis [19] for cerebral NADH under baseline conditions. The vectorial sum of the 4 computed lifetime components are displayed by solid arrows. Each vector is colored to match the corresponding component, and their lengths are scaled by their corresponding fractional fluorescence. The fractionally-weighted vectorial sum localizes in the same area of the universal circle as the computed phasor coordinates from *in vivo* cerebral NADH measurements, suggesting consistency between our nonlinear fitting and phasor analysis methods.

Fig 2 displays intensity changes and phasor results of cerebral NADH measured before and after pharmacological manipulation. For each measurement, the average values for g(ω) and s(ω) were computed over all pixels in the full field of view (~200 μm) after masking out cerebral blood vessels, yielding a single phasor coordinate pair for each measurement. With a minimum of 3 measurement locations per animal and minimum 3 animals per manipulation, at least 9 data points exist for each pharmacological manipulation. The lower plots focus on the regions of the phasor plot corresponding to cerebral NADH and display the standard deviational ellipses [36] of our phasor results. Standard deviational ellipses delineate 2-dimensional bivariate dispersion and were computed as described in Yuill, et al [36,37]. Ellipses and data point colors correspond to colors in Fig 2A to indicate the pharmacological reagent and inhibited metabolic process. Fig 2E displays the results of the Hotelling t-squared statistical tests for each pharmacological inhibitor to assess whether the phasor coordinates vary significantly from baseline measurements.

Glycolytic inhibition was induced by either 2-deoxyglucose (2DG) or iodoacetic acid (IAA). For these experiments, downstream oxidative metabolism was sustained by administering 1 mM sodium pyruvate alongside the pharmacological inhibitors. Phasor measurements for glycolysis-inhibited cortical tissue are not distinct from those of baseline phasor values, as illustrated by the low t-squared statistic and corresponding p-values, and also reflected by the substantial overlap of standard deviational ellipses in Fig 2C and 2D. All other pharmacological manipulations affect metabolic processes occurring in the mitochondria and were found to induce significantly different phasor coordinates and intensity changes relative to baseline (p-value < 0.05).

When applying phasor analysis to frequency-domain-based lifetime measurements, the modulation frequency of excitation light affects the phasor plot by mapping lifetimes on different regions of the phasor universal circle. With higher modulation frequencies, shorter lifetimes become more spread out over the right half of the universal circle, while longer lifetimes become more compressed on the left side. If the lifetimes of fluorophores are well-known in advance, the optimal modulation frequency can be computed to enable maximal separation on the polar plots [22,29]. For time-domain lifetime measurements, similar practices have been implemented, in which the phasor values are computed at integral harmonics of the laser repetition frequency. Fig 2C and 2D show computed results for the first (k = 1) and second (k = 2) harmonics, ω = k* 2π* f, of the laser repetition frequency (f = 80 MHz). 2^nd^ harmonic phasor coordinates of our *in vivo* cerebral NADH observations appear shifted to the left and standard deviational ellipses appear oriented differently in shape and distance from one another. However, Hotelling t-squared analysis revealed that significant separability from baseline was the same for first and second harmonic phasor values for all pharmacological inhibitions.

All inhibitions of metabolic processes occurring within mitochondria cause the centroids of phasor clusters to migrate in the general direction of NADH solution (free NADH), although the separability from baseline and spatial distribution varies for each process. Relative to baseline measurements, each pharmacological inhibitor induces shifts of the phasor values with a different magnitude and direction. The separations of clusters on the phasor plot suggests unique variations in the relative abundance of enzyme-bound NADH formulations that vary with particular pharmacological agents, consistent with our nonlinear fitting analyses [19]. The results reinforce the repeated assertion that NADH exists as a distribution of different enzymatic formulations [17,38]. Inhibitions to the electron transport chain induced the most pronounced separations from baseline, with corresponding phasor clusters shifted more toward free NADH. This is consistent with our previous analyses, which showed that free NADH contributes more to the fractional fluorescence after inhibition of the electron transport chain. While additional investigation is required, these observations suggest that the enzymes associated with the electron transport chain (likely complex I, NADH dehydrogenase) constitute the most prevalent enzymatic formulation in cortical astrocytes and neurons. In general, contributions to autofluorescence from spectrally identical NADPH, also require consideration and could confound interpretation of metabolic activity [17]. As it does not participate in glycolysis or oxidative metabolism, we contend that NADPH is insensitive to the metabolic perturbations induced in this study [16,39,40]. NADPH would therefore contribute to the observed fluorescence as a constant background signal with unvarying fluorescence lifetime and amplitude. When applying phasor analysis, this constant offset before and after metabolic perturbation would not confound analysis of separability. Importantly, we also observed a considerable increase in NADH intensity when blocking the electron transport chain. An increase in NADH intensity could correspond to greater prevalence of protein-bound NADH species, which display longer lifetimes and higher quantum yields. However, this phenomenon is not consistent with our observed shifts of phasor representations toward free NADH in Fig 2C and 2D, nor is it supported by reported changes in lifetime values from experiments involving rotenone [17]. Both the increase in NADH intensity and corresponding relative increase in shorter lifetime component are most likely the result of a greater overall amount of NADH.

Using the Statistics and Machine Learning Toolbox in Matlab, classification algorithms were developed to evaluate whether phasor analysis of NADH FLIM data can accurately identify metabolic perturbations associated with neuropathological conditions. Computed phasor values and NADH intensity changes were utilized as training data, while measurements of BMI-induced focal seizure activity served as test data. Data from Fig 2B–2D (phasor coordinates and changes in NADH intensity from baseline (g(ω), s(ω), and ΔI)), were used as input parameters to develop linear discriminant analysis (LDA), K-nearest neighbor (KNN), and naive Bayes (NB) classification models [31]. Table 1 summarizes results from the 3-parameter models, developed using both first and second harmonic phasor results. For comparison, Table 1 also displays previously-reported results from the 6-parameter models curve fitting metrics [19]. K-fold cross validation error was computed by repeatedly testing subsets of the training data as input for the models and found to be slightly better for both phasor-based models. Both 3-parameter models performed similarly for classifying BMI-induced seizures as disruptions to the electron transport chain and comparable to the 6-parameter model. The higher prediction accuracy and reduction in dimensionality constitute a substantial improvement compared to the previous 6-dimensional models.

**Table 1:** Statistical classification model results.

Classification Model		Nonlinear fitting	Phasor Analysis Harmonic 1	Phasor Analysis Harmonic 2
**# Parameters**		**6**	**3**	**3**
**LDA**	**K-fold cross- validation**	67%	76%	76%
	**BMI Prediction**	92%	92%	92%
**KNN**	**K-fold cross- validation**	71%	78%	73%
	**BMI Prediction**	92%	100%	100%
**Naive Bayes**	**K-fold cross-validation**	73%	78%	78%
	**BMI Prediction**	92%	92%	92%

K-fold cross validation accuracy and classification accuracy for focal seizure test-data of classification models based on nonlinear fitting, phasor analysis harmonic 1 and/or phasor analysis harmonic 2.

## Conclusion

Phasor analysis is a powerful, elegant tool, which is particularly useful for resolving variations in the relative proportions of 2 single exponential fluorophores with well-separated lifetimes, such as in FRET experiments [22,23,29]. Compared to conventional curve fitting methods for FLIM analysis, the phasor technique provides a computationally straightforward approach without assumptions about underlying chemical interactions (e.g., number of fluorescent decay components) and easy to visualize method for resolving variations in fluorescence lifetimes. Here, we demonstrated that the method can be applied to endogenous cerebral NADH fluorescence lifetime measurements to identify alterations in mitochondrial processes, such as the Krebs’ cycle, oxidative phosphorylation, and respiration. Variations in cytosolic glycolysis appear too subtle to resolve from baseline activity using the phasor approach, as indicated by the lack of statistically significant differences from baseline and poor separability of 2DG- and IAA-induced manipulations in Fig 2B and 2C. Given that the majority of NADH fluorescence is reportedly mitochondrial in origin [41], and the significant destructive role played by mitochondrial dysfunction in numerous neurological disorders [42], these results demonstrate that combining phasor analysis and two-photon fluorescence lifetime imaging enhances the potential utility of endogenous NADH fluorescence as a prospective biomarker for cerebral pathologies. Collectively, coupling nondestructive imaging of endogenous NADH with FLIM together with computationally-elegant phasor analysis and statistical classification models demonstrates considerable potential as a powerful, objective analytical tool that can potentially enable real time NADH FLIM measurements for reliable disease diagnosis and staging.

## Supporting information

S1 File Gomez_PLOS_supplementary_file. (PDF)
