# Supplementary material for: Phasor analysis of NADH FLIM identifies pharmacological disruptions to mitochondrial metabolic processes in the rodent cerebral cortex
Source: PLoS One. 2018 Mar 21;13(3):e0194578. doi: 10.1371/journal.pone.0194578 (PMC5862490; doi:10.1371/journal.pone.0194578)
Supplement: S1 File — (PDF) [file pone.0194578.s001.pdf]

Calibration of phasor data with a reference standard.

For a fluorophore whose fluorescence is modeled as a single exponential decay the computed phasor, denoted here as *Ph{}*, is expressed as:

$$Ph\{I(t)\} = \frac{\mathcal{F}\{I(t)\}}{\int_0^{\infty} I(t)\,\mathrm{dt}} = \frac{\int_{-\infty}^{\infty} I_0 e^{-\frac{t}{\tau}} e^{-i\omega t}\,dt}{\int_0^{\infty} I_0 e^{-\frac{t}{\tau}}\,dt}$$

$$=\frac{1}{1+j\omega\tau} = \frac{1-j\omega\tau}{1+(\omega\tau)^2}$$

$$= g - js$$

And a fluorophore with multiexponential lifetime decay is expressed as:

$$Ph\{I(t)\} = \frac{\mathcal{F}\{I(t)\}}{\int_0^{\infty} I(t)\,dt} = \frac{\mathcal{F}\left\{\sum_{i=1}^{N} \alpha_i e^{-\frac{t}{\tau_i}}\right\}}{\int_0^{\infty} \sum_{i=1}^{N} \alpha_i e^{-\frac{t}{\tau_i}}\,dt} = \sum_{i=1}^{N} \frac{1}{\sum_{k=1}^{N} \alpha_k \tau_k} \frac{\alpha_i \tau_i}{1+\omega\tau_i}$$

$$= \sum_{i=1}^{N} \frac{\alpha_i \tau_i}{\sum_{k=1}^{N} \alpha_k \tau_k} \frac{1}{1+\omega\tau_i} = \sum_{i=1}^{N} f_i \left(\frac{1}{1+(\omega\tau_i)^2} - \frac{j\omega\tau_i}{1+(\omega\tau_i)^2}\right)$$

$$g = \sum_{i=1}^{N} f_i \left(\frac{1}{1+(\omega\tau_i)^2}\right) = \sum_{i=1}^{N} f_i g_i \qquad s = \sum_{i=1}^{N} f_i \left(\frac{\omega\tau_i}{1+(\omega\tau_i)^2}\right) = \sum_{i=1}^{N} f_i s_i$$

, where f_i, g_i, and s_i denote each exponential term's fractional fluorescence and computed phasor coordinate along the universal phasor circle [1].

Experimental observations of fluorescence lifetime include contributions from the instrument response function (IRF), defined as the imaging system's detected pulse shape in response to an infinitesimally short impulse of light. Similar to nonlinear curve-fitting analysis, phasor analysis requires a calibration procedure to account for this.

The experimentally-detected time-resolved fluorescence decay, I_{exp} *(t),* can be expressed as

$$I_{exp}(t) = I_{the}(t) \ * IRF(t)$$

Where I_{the} *(t)* describes the fluorophore's theoretical fluorescence decay and *IRF(t)* denotes the IRF.

Given these relationships:

$$\int_0^\infty I_{exp}(t)\,\mathrm{dt} = \int_0^\infty \left(I_{the}(t) \ * IRF(t)\right) \ \mathrm{dt} = \int_0^\infty I_{the}(t)\,\mathrm{dt} \cdot \int_0^\infty IRF(t)\,\mathrm{dt}$$

$$\mathcal{F}\{I_{exp}(t)\} = \mathcal{F}\{I_{the}(t) \ * IRF(t)\} = \mathcal{F}\{I_{the}(t)\} \cdot \mathcal{F}\{IRF(t)\}$$

Ph{I_{the}*(t)}* can easily be related to Ph{I_{exp}*(t)}* through a procedure that accounts for the system's IRF:

$$Ph\{I_{the}(t)\} = \ Normalized\,\mathcal{F}\{I_{exp}(t)\} = g_{exp} + js_{exp} = \frac{\mathcal{F}\{I_{exp}(t)\}}{\int_0^\infty I_{exp}(t)\,\mathrm{dt}}$$

$$= \frac{\mathcal{F}\{I_{the}(t)\} \cdot \mathcal{F}\{IRF(t)\}}{\int_0^\infty I_{the}(t)\,\mathrm{dt} \cdot \int_0^\infty IRF(t)\,\mathrm{dt}} = (g_{the} + js_{the})(g_{IRF} + js_{IRF})$$

The relationship can easily be formalized in matrix notation as:

$$Ph\{I_{exp}(t)\} = (g_{exp} + js_{exp}) = (g_{the} + js_{the})(g_{IRF} + js_{IRF})$$

$$= (g_{the}g_{IRF} - s_{the}s_{IRF}) \ + \ j(s_{the}g_{IRF} + g_{the}s_{IRF})$$

$$\begin{bmatrix} g_{exp} \\ s_{exp} \end{bmatrix} = \begin{bmatrix} g_{IRF} & -s_{IRF} \\ s_{IRF} & g_{IRF} \end{bmatrix} \begin{bmatrix} g_{the} \\ s_{the} \end{bmatrix}$$

With knowledge of g_{irf} and s_{irf}, we can easily solve for the phasor coordinates of I_{the}*(t)*:

$$\begin{bmatrix} g_{the} \\ s_{the} \end{bmatrix} = \frac{1}{g_{IRF}^2 + s_{IRF}^2} \begin{bmatrix} g_{IRF} & s_{IRF} \\ -s_{IRF} & g_{IRF} \end{bmatrix} \begin{bmatrix} g_{exp} \\ s_{exp} \end{bmatrix}$$

We utilized our measurements of NADH dissolved in saline as a reference standard to compute g_{the} and s_{the}.. Previously, we determined that using NADH solution for calculating IRF(t) worked better than other widely-utilized reference standards such as second harmonic generation measurements of collagen because it avoided wavelength-dependent variations in IRF(t). The IRF(t) was found to vary considerably with excitation wavelengths, as we observed by detecting faint reflections from a non-fluorescent reflective surface.

As described in our previous report, we computed the IRF using an iterative procedure. Nonlinear fits were repeatedly performed on experimental measurements of NADH solution, where each successive iteration utilized the IRF calculated in the previous iteration. From these iterative calculations, *IRF(t)*, as well as amplitude and lifetime values for $I_{the}(t)$ for NADH solution, were chosen from the iteration that yielded the smallest fitting error [2].

Measurements of NADH solution were collected in advance of each experiment reported here. For these phasor computations, $I_{the}(t)$ was taken as the amplitude and lifetime values averaged over all measurements of NADH solution in this study:

$$NADH\ solution: \quad I_{NADH}(t) = 0.91e^{-\frac{t}{0.401}} + 0.09e^{-\frac{t}{1.174}}$$

$$f_1 = 0.78 \ \tau_1 = 0.401; \qquad f_2 = 0.22 \quad \tau_2 = 1.174$$

These values were used to compute the standard phasor coordinates of NADH solution.

$$Ph\{I_{NADH}(t)\} = f_1 \left(\frac{1 + j\omega\tau_1}{1 + \omega\tau_1{}^2} \right) + f_2 \left(\frac{1 + j\omega\tau_2}{1 + \omega\tau_2{}^2} \right)$$

$$Ph\{I_{NADH}(t)\} = 0.914 + j0.246$$

As seen in fig S1 below, our algorithms results are consistent with the recently-employed phasor analysis included in commercial SPCIMage software from Becker & Hickl, as well as published reports [3].

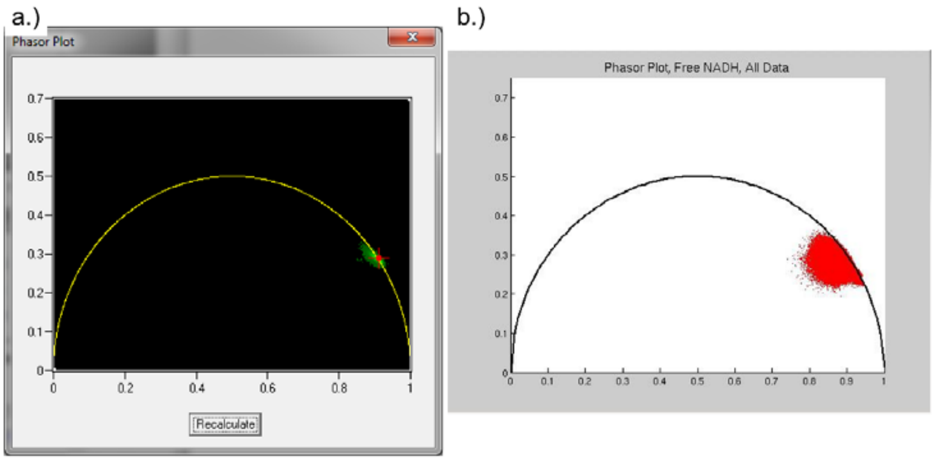

Figure S1. Phasor computations for a cuvette of NADH dissolved in saline as computed by commercial software (SPCIMage, Becker & Hickl GmbH), and our custom analysis routines

For each experiment, g_{irf} and s_{irf} were computed using the standard phasor coordinates and phasor coordinates of the corresponding experimental measurement for NADH solution $g_{exp,\ NADH}$ $s_{exp,\ NADH}$. These values were then used to compute for g_{the} s_{the} of our *in vivo* NADH measurements.

$$\begin{bmatrix} g_{irf} \\ s_{irf} \end{bmatrix} = \frac{1}{g_{exp,NADH}^2 + s_{exp,NADH}^2} \begin{bmatrix} g_{\mathrm{exp},NADH} & s_{exp,NADH} \\ -s_{exp,NADH} & g_{exp,NADH} \end{bmatrix} \begin{bmatrix} g_{exp} \\ s_{exp} \end{bmatrix}$$

After calibration, our *in vivo* observations of cerebral NADH appear to localize in the same region as *in vitro* observations of neurons and neural progenitor stem cells [4].

REFERENCES

1. M. A. Digman, V. R. Caiolfa, M. Zamai, and E. Gratton, "The phasor approach to fluorescence lifetime imaging analysis," Biophys. J. **94**, L14–L16 (2008).
2. M. A. Yaseen, S. Sakadžić, W. Wu, W. Becker, K. A. Kasischke, and D. A. Boas, "In vivo imaging of cerebral energy metabolism with two-photon fluorescence lifetime microscopy of NADH," Biomed. Opt. Express **4**, 307–321 (2013).
3. C. Stringari, A. Cinquin, O. Cinquin, M. Digman, P. J. Donovan, and E. Gratton, "Phasor approach to fluorescence lifetime microscopy distinguishes different metabolic states of germ cells in a live tissue," Proc. Natl. Acad. Sci. U. S. A. **108**, 13582–13587 (2011).
4. C. Stringari, J. L. Nourse, L. A. Flanagan, and E. Gratton, "Phasor Fluorescence Lifetime Microscopy of Free and Protein-Bound NADH Reveals Neural Stem Cell Differentiation Potential," PLoS ONE **7**, e48014 (2012).
